# Overview of HPCAT and capabilities for studying minerals and various other materials at high-pressure conditions

**DOI:** 10.1007/s00269-022-01209-2

**Published:** 2022-08-15

**Authors:** Arunkumar Bommannavar, Paul Chow, Rich Ferry, Rostislav Hrubiak, Freda Humble, Curtis Kenney-Benson, Mingda Lv, Yue Meng, Changyong Park, Dmitry Popov, Eric Rod, Maddury Somayazulu, Guoyin Shen, Dean Smith, Jesse Smith, Yuming Xiao, Nenad Velisavljevic

**Affiliations:** 1 High Pressure Collaborative Access Team (HPCAT), X-Ray Science Division, Argonne National Laboratory, Lemont, IL 60439 USA; 2 Physics Division, Lawrence Livermore National Laboratory, Livermore, CA 94550 USA

**Keywords:** HPCAT, Pressure, Synchrotron

## Abstract

High-Pressure Collaborative Access Team (HPCAT) is a synchrotron-based facility located at the Advanced Photon Source (APS). With four online experimental stations and various offline capabilities, HPCAT is focused on providing synchrotron x-ray capabilities for high pressure and temperature research and supporting a broad user community. Overall, the array of online/offline capabilities is described, including some of the recent developments for remote user support and the concomitant impact of the current pandemic. General overview of work done at HPCAT and with a focus on some of the minerals relevant work and supporting capabilities is also discussed. With the impending APS-Upgrade (APS-U), there is a considerable effort within HPCAT to improve and add capabilities. These are summarized briefly for each of the end-stations.

## Introduction

Over the last five decades, high-pressure research has become synonymous with x-ray light sources (Bassett 2009). Large scale x-ray light sources, in particular, synchrotrons and now more recently also x-ray free electron lasers (XFEL), provide the necessary high brightness and high energy beams for probing material under a range of pressure (P), temperature (T), and strain rate ($\dot{\upvarepsilon }$) conditions. Some of the earliest high-pressure research at synchrotron sources was vital in providing P–T and volume (V) compression (i.e., density change or equation of state) data and establishing P–T phase diagram for a range of materials. Using x-ray diffraction (XRD) as the primary technique, in the earliest stages, crystal structure information and subsequent solid–solid and solid-melt type transitions could be measured as the material is subjected to high P–T conditions. Scientific and technological impact from these measurements was realized immediately and in turn this led to further growth and expansion of high-pressure research at the light sources. Subsequently, with the evolution of high-pressure research platform and light sources the range of P–T conditions and x-ray diagnostics has expanded significantly. Today, across the globe, there are numerous beamlines and sectors, dedicated to high-pressure research (Stan et al. 2018) featured at large-scale light sources. However, it is important to note, not all high-pressure facilities are the same. The differences are not only with respect to scientific scope, but they are also rooted in the institutional agreements, arrangements with the funding sources (and this varies across countries), and various other drivers relating to the user base etc.

HPCAT, located at the Advanced Photon Source (APS) at Argonne National Laboratory, outside of the city of Chicago in the USA, was established in the early 2000s. APS was introduced as the third-generation x-ray synchrotron source and received its first light in the late 1990s, after which HPCAT became one of the earliest beamline sectors to come online. Currently, along with the Geo Soil and Enviro (GSECARS)—also featured in this issue—and the Dynamic Compression Sector (DCS), dedicated to high strain rate shock studies, HPCAT is one of three dedicated sectors (either in full or significant part), to studies of materials under extreme P–T-$\dot{\upvarepsilon }$ conditions (The Advanced Photon Source 2021). As the name indicates, HPCAT is based on the Collaborative Access Team (CAT) model. The CAT model was introduced in the USA light sources almost 30 years ago, as a process for users with a common interest to form consortia or teams and operate facilities at light sources. Although CATs are funded and operated separately from the host synchrotron facility, there is a mutual benefit in that the host facility oversees operations of the storage ring and delivering x-rays into the experimental stations, while the CAT member brings special expertise (e.g., in case of HPCAT the expertise is in high-pressure devices). Under the mutual benefit umbrella, the CAT members retain a certain portion of beamtime, as agreed with the host institution and funding agencies, while at least 25% is offered to the broader user community and anyone who has a successful research proposal (reviewed by a scientific committee that is independent of CAT). Although some of the logistical and operational infrastructure nuances can be viewed as beyond the scientific research scope, it is still rather important to share as it steers the work that is performed at each beamline/station, beamtime allocation access (or percentage breakdown in available shifts), and other impacts.

**Fig. 1:**
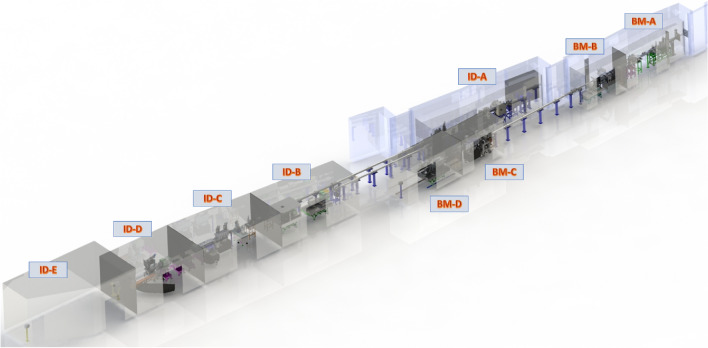
Schematic layout of HPCAT, showing the various experimental and optics stations

Initially, HPCAT was started as a single beamline and over the last ~ 20 years has grown into five experimental stations and various offline laboratory support facilities, located in sector-16 of the APS (Fig. 1). As previously noted, the majority of the early high-pressure work at light sources was focused on XRD type measurements with diamond anvil cell (DAC) pressure device. However, as the light sources and associated optics, detectors, and storage ring timing/bunch modes evolved, the array of x-ray measurements that could be performed with DAC also increased substantially (Bassett 2009) (Shen and Mao 2016). With APS overseeing the storage ring and delivery of the x-ray beam, HPCAT sector responsibility begins at the entrance of the beam into the first optics enclosure (FOE) stations, labeled as BM-A, BM-C, and ID-A, in Fig. 1.

HPCAT can be viewed as two independent branches, which can be traced back to the delivery of the x-ray beam via canted undulators/insertion device (ID branch) or the bending magnet (BM branch). Optics stations primarily include beam shutters, shielding, and Radiation Safety Systems (RSS), as well as various crystal monochromators, as discussed in further detail in the following sections. In addition to optics stations, the experimental end-stations, BM-B, BM-D, ID-B, ID-D and ID-E, are operated simultaneously and provide various x-ray capabilities for spectroscopy, diffraction/scattering, and imaging measurements. Having a canted undulator for the ID branch allows for the independent operation of ID-B and ID-D/E stations. Although the introduction of high-pressure work at light sources centered initially around DAC, in ensuing time, larger platforms were also established as dedicated user capabilities (McMillan 2005). Today, the HPCAT facility provides:

- **16-BM-B**: multi-array capabilities, including large volume Paris-Edinburg (PE) press (sample size up to ~ 1 mm diameter and 1 mm thick disk) and white-beam Laue x-ray microscopy. The PE press, which can generate conditions up to ~ 8 GPa and ~ 2500 K, can be used to obtain in situ viscosity measurements of melts/liquids, longitudinal and shear elastic moduli from a coupled piezo-transducer ultrasonic setup, and relative changes in thermal/electrical conductivity. The white-beam Laue technique is tailored for measurements with diamond anvil cell (DAC) and provides cutting-edge measurements of structural deformation and transformation mechanism of materials during high-pressure loading/heating.
- **16-ID-B and 16-BM-D**: x-ray diffraction and in situ laser and resistive heating – measurements can be performed over a broad range of pressures (ambient to > 500 GPa) and temperatures (~ 10 K up to 4000 + K) with resulting pressure–volume-temperature data being applied for the equation of state models and multiphase P–T phase diagram development, understanding the kinetics of phase transitions and structural stability, and determining solid-melt boundary. In addition, x-ray absorption spectroscopy (XANES and XAFS) can be performed in tandem with diffraction in 16BM-D which is a unique capability that helps unravel the microscopic mechanism of pressure-induced changes.
- **16-ID-D**: x-ray Raman spectroscopy, x-ray emission spectroscopy, inelastic x-ray scattering – provides key measurements of bonding and electronic structure for understanding material stability at high pressure–temperature conditions.
- **16-ID-E**: an approved x-ray enclosure fielding special request and unique measurements. For example, in an effort toward brining DCS online this station was used for the demonstration of the first gun-shock experiment measurement with APS x-ray light source (Gupta et al. 2012).

With our operations model, which is tailored to support our CAT stakeholders and the broader user community, users typically come to HPCAT with experiments and DAC prepared in advance. However, HPCAT provides various offline capabilities and laboratory space to accommodate users who require additional sample preparation while onsite. Our user support is vital in the case of experiments with the PE press, where the complete sample assembly is specifically designed and machined per user request. Details pertaining to available techniques are discussed in the following sections and quick reference material, including updates and published examples, are also posted on HPCAT website (HPCAT at the Advanced Photon Source 2021).

As with everyone around the globe, HPCAT operations were also significantly impacted by the pandemic over the last two years. In a move to deal with the pandemic user access to HPCAT and APS was closed in March of 2019. Subsequently, in the following months, keeping the immediate needs in perspective, we considered establishing remote-type operations. In months following the initial closure, HPCAT implemented additional infrastructure, protocols, and components that would allow mail-in of experimental equipment which would be setup at the beamline by HPCAT staff following which, the full remote control of the experimental setup is handed over to the remote-user. From the onset, it was clearly defined that the HPCAT staff was not going to perform the experiments for others, but rather our effort would be in providing onsite setup effort and then having the user perform experiments, as would be the case under regular pre-pandemic operations. To aid in the new remote effort, HPCAT installed cameras in all stations that allow for a remote view of the experimental setup. The APS and Argonne-led initiative provided network and remote login infrastructure (No Machine NX-Server) for the users to be able to connect and take over full control of experimental setup, including sample stages, opening shutters for x-ray beam, adjusting focusing, controlling sample pressure and all other equipment. To increase throughput and continuous experimental measurements, a new pneumatic-DAC-changer was developed in house and installed at all stations. In the case of PE press, a new syringe pump was installed, which allowed full remote control of pressure increase/decrease, while also providing better pressure stability on the sample during prolonged measurements at set P–T conditions. Finally, once measurement begins, the remote user can access and transfer data via the APS Globus data storage and management system. Although the majority of our users are currently coming again onsite, the remote operations setup has been kept as an alternative and available when requested.

In addition to the recent effort in establishing remote-operations support, in the last three years HPCAT has also been preparing for the upcoming upgrade to the APS storage ring (The APS Upgrade: Building a Brighter Future 2021). Currently, APS is in the final stages of preparing to undergo a significant upgrade (APS-U) to the storage ring and installation of a new seven multiband achromat (MBA) lattice, which is expected to provide a significant improvement to brightness, coherent flux, and overall performance of the ring. As part of the APS-U, most of the individual experimental stations/sectors are also preparing for the scheduled shutdown starting in April of 2023. HPCAT has been working diligently in preparing to upgrade various components as well, including an upgrade to the canted undulator source, redesign or updates to the monochromators and various optical components, and purchase of new detectors, stages, and other experimental equipment. The overall discussion of HPCAT upgrade plan is beyond the scope of this publication, however, some general information is required so that the reader can have relevant background and foresight to gauge the impact on our current capabilities and operations. For example, on BM branch, it is expected that the current two simultaneously operational beamlines, BM-B and BM-D, will need to be restructured and post APS-U would run in series. Although the full array of experimental capabilities that are being offered to users currently will not decrease, we do expect to undergo a significant rearrangement of equipment and redesign of the experimental stations. Overall, HPCAT planning for the upgrade is being performed in stages. First, we are committed to retaining all the experimental capabilities currently offered, and hopefully with significant improvements. We are also working on making sure that those capabilities are available as soon as the APS-U is completed. In the second stage, our focus is on introducing new capabilities that can only be enabled with APS-U beam, for example, higher brightness and coherence would allow measurements with improved spatial and temporal resolution. We expect to have improvements in all the experimental stations and the following sections will provide an overview of current HPCAT capabilities and possibilities in the near term and with APS-U.

### Beamline 16-ID-D: Spectroscopy, inelastic X-ray scattering and other measurements

Sector 16ID-D of HPCAT uses mainly spectroscopic and inelastic x-ray scattering methods to study electronic and magnetic properties of materials under high pressure, typically in diamond anvil cells (DACs). The beamline is equipped with a liquid N_2_ cooled Si(111) double crystal monochromator (DCM) which provides x-rays in the range of 4.5–37 keV. Current available techniques include x-ray emission spectroscopy (XES), inelastic x-ray scattering (IXS) and nuclear resonant scattering (NRS). Due to the small inelastic scattering cross-section, all the available techniques require high incident flux and large solid angle collection. The x-ray beam is focused to ~ 3(V) × 6(H) μm FWHM in size at the sample position using 320 mm (V) × 400 mm (H) long KB mirrors.

**Fig. 2:**
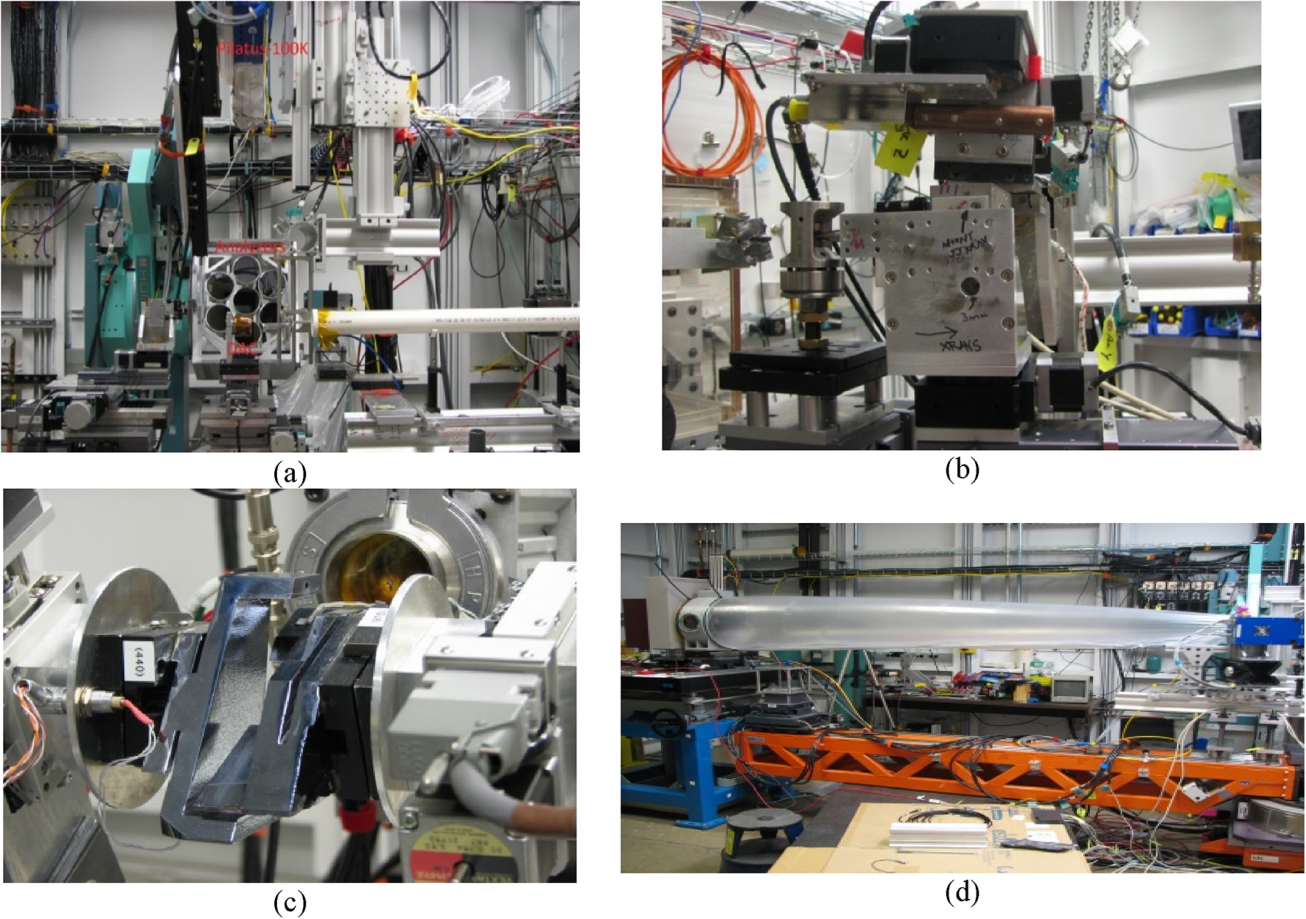
**a** 7-element XES spectrometer using a Pilatus 100 K as detector **b** IXS spectrometer showing a 7 mm focal length polycapillary lens inserted into a panoramic DAC **c** High-resolution monochromator for ^57^Fe NRS **d** HP SAXS setup using Pilatus 100 K for WAXS and Mar345 image plate for SAXS measurements

Since the first application under high pressure in the late 1990s, XES has been widely used to study pressure-induced spin transitions of Fe-containing minerals and other materials by measuring satellite peaks of the K_β1,3_ line of 3*d* transition metal and the L_γ1_ line of 4*f* elements (Xiao et al. 2016). Figure 2a shows the one-meter Rowland circle XES spectrometer in the vertical scattering geometry. The XES spectrometer consists of up to seven 4″ spherical Si analyzers and a Pilatus 100 K as the detector. Using the same experimental setup, resonance XES (RXES) and partial fluorescence yield x-ray absorption spectroscopy can be performed to obtain information on intra-atomic multiplet coupling, electron correlation and inter-atomic hybridization of samples under high pressure.

Inelastic x-ray scattering (IXS) covers several photon-in photon-out techniques. HPCAT has an IXS spectrometer for the study of electronic excitations at low momentum transfer and core-electron excitations (x-ray Raman scattering) at a larger scattering angle with ~ 1.4 eV energy resolution. Post-sample collimation is important for high-pressure IXS experiments because of background scattering from other DAC components (diamond anvils, Be gasket etc.). Since 2016, a full polycapillary lens has been used in the IXS spectrometer at HPCAT to collect sample scattering signals from a large solid angle and minimize background scattering, as shown in Fig. 2b (Chow et al. 2015). With a much-improved signal-to-noise ratio and focus size of a few μm, XRS experiments above Mbar have been routinely done at HPCAT in recent years. For example, Lee et al. have measured XRS spectra for SiO_2_ up to 1.6 Mbar and revealed the potential emergence of quadrupled coordinated oxygen (oxygen quad-cluster) (Lee et al. 2019).

Based on the high brightness of 3rd generation synchrotron sources, the development of tunable monochromators with meV resolution and fast detectors with nano-second time resolution, nuclear resonant scattering (NRS) has become a relatively new spectroscopic method for high-pressure research since 2000. NRS has isotope selectivity which is very helpful for high-pressure experiments by suppressing background scattering from other DAC components. NRS can be divided into two methods: nuclear resonant inelastic x-ray scattering (NRIXS) and nuclear forward scattering (NFS). Partial phonon density of states (PDOS) can be derived from experimental NRIXS spectra to extract important dynamic, thermodynamic and elastic information, such as the vibrational entropy, the Debye temperature, and sound velocities. Hyperfine interaction parameters can be obtained from NFS spectra to give information on the spin state, oxidation state and magnetic structure of samples. HPCAT has a high-resolution monochromator (HRM) for ^57^Fe NRS experiment at 14.413 keV (shown in Fig. 2c). Klein et al. has used a suite of experimental techniques (XES and parts of NFS experiments were done at 16 ID-D) to report a pressure-induced phase transition in the frustrated Kagome material jarosite at ~ 45GPa, which leads to the disappearance of magnetic order (Klein et al. 2020).

In addition to experiments using the three main techniques mentioned above, 16 ID-D has conducted many other measurements such as imaging and optics tests in recent years. Since 2019, 16 ID-D has started to build and commission a setup, shown in Fig. 2d, for high-pressure small angle x-ray scattering (HP-SAXS). In the setup, a Pilatus 100 K is used for wide-angle x-ray scattering (WAXS) and a MAR345 image plate ~ 3 m from sample is used for SAXS. Meng et al. combined HP XRD and SAXS to characterize the structural and morphological changes in PbS and PbSe nanocrystals and demonstrated that the applied pressure induced a reversible atomic phase transition of the nanocrystals under hydrostatic pressure (Meng et al. 2021).

Over the last few years, 16ID-D has refined WAXS/SAXS measurements in a DAC (see Fig. 2d for layout) as well as IXS measurements. Use of poly-capillary and mono-capillary lenses to increase spatial filtering (Fig. 2b), as well as enhancements in analyzer and monochromator and plans to incorporate a custom-built cryostat for XRS measurements are planned post-upgrade. Similarly, the use of long focal length CRLs for focusing is expected to increase the S/N ratio in SAXS measurements. The spectroscopy program in 16ID-D is expected to benefit from the inclusion of a new DCM/DMM in the 16ID-C station while phasing out the existing DCM in the FOE hutch during the upgrade. The addition of the DMM offers higher flux for performing pump-probe XRD and XAS studies that are expected to open a new vista in extreme conditions science. In addition, plans for the now-empty ID-E hutch include setting up a multi-purpose instrument with high positional accuracy and reproducibility (sphere of confusion < 1 μm^3^) and a sub- μm focal spot to enable diffraction tomography, coherent diffraction imaging, etc. Optics simulations have suggested that double focusing with the CRL lenses and table-top pre-engineered KB mirrors can yield 200–500 nm focal spots with a nominal size of 1–2 μm at the 90% width both with the monochromatic beams (DCM) as well as the large bandpass beam (DMM). This capability is planned to be exploited in performing powder and multi-crystal diffraction at TPa pressures as well as including other platforms for conducting diffraction studies.

### Beamline 16-ID-B: laser heating and micro-diffraction at extreme pressure–temperature conditions

The outboard beam from the canted undulator sources (3.3 Undulator A) at HPCAT is used to independently operate the ID-B hutch serviced by a horizontally deflecting monochromator that allows spatial separation of the ID-D and ID-B end-stations, as well as an independent selection of undulator harmonics and operating wavelengths. The branching monochromator is equipped with three crystal sets that allow tuning between 18 and 50 keV(cf. Table 1). The monochromatic beam that enters the ID-B hutch services two tables in tandem. The first of them is a general purpose (GP) table that enables high-pressure micro-diffraction while the second one is the laser heating (LH) table.

**Table 1:** Primary components for the ID-B beamline and GP table end station. A nominal configuration using the Ge(111) monochromator at 29.200 keV and the large KB mirror assembly provides a measured flux of 1.4 × 10^12^ photons/second at the sample position

Source	Insertion device	APS Undulator A
Monochromator	Crystal pairs	Ge(111), Si(111), Si(220)
	Energy range	~ 18–50 keV
Focusing	320 mm × 320 mm (v × h) KB mirrors	2 × 5 μm^2^ (v × h) focal spot, FWHM
	200 mm × 100 mm (v × h) KB mirrors	1 × 2 μm^2^ (v × h) focal spot, FWHM
Clean-up	Tungsten pinholes	10–150 + μm diameter
Hybrid sample stack	Hexapod (high load)	40 kg load capacity
	Linear stack (compact size)	5 kg load capacity
Detector	PILATUS 1 M-F	450 um Si, 125 Hz

The guiding principle for the operation and development of the GP table is to offer maximum flexibility with the experimental configuration while at the same time maintaining the highest data quality and scientific productivity. The flexible beamline configuration—together with online and supporting equipment—enables powder, polycrystalline, multigrain, and single-crystal diffraction both in axial and/or radial geometries. Cryogenic cooling and resistive heating facilitate temperature-dependent high-pressure studies over an approximate range of 5–1000 K. Remote, programmable, double-sided pressure control enable time-dependent compression and decompression (or similarly, variable strain rate) measurements. Fast strain rates are possible with piezo-driven dynamic DACs and the gas membrane system. Online optical assemblies allow real-time sample visualization and fluorescence pressure measurements.

Users can choose among three monochromator crystal pairs with varying scattering powers and corresponding energy ranges, depending on the specific experimental needs for photon flux, diffraction resolution, spatial resolution, and/or detector efficiency. Focusing is achieved using (any one of two) Kirkpatrick-Baez (KB) mirror assemblies. The larger mirror assembly accepts a typical incident beam of 0.500 mm × 0.500 mm (v x h) at ~ 30 keV for maximum flux. The smaller mirror assembly accepts a typical incident beam of 0.300 mm × 0.150 mm (v x h), yielding a much smaller focal spot at the cost of offering roughly 20% of the flux compared to the large assembly. Circular tungsten clean-up apertures are used to eliminate weak scattering arising from transmission and imperfections from the mirrors. In an effort to accommodate a wide range of sample assemblies of various dimensions and mass, the GP table uses a hybrid sample stack that includes a hexapod and high-precision XPS stages. For most experiments, a compact sub-assembly of stages mounted on top of the hexapod is used for sample positioning and scanning. For sample assemblies exceeding 5 kg (e.g., vacuum enclosures required for low- and high-T experiments), the compact assembly is removed, and a hexapod is used for sample positioning and scanning. A beamstop with an integrated diode enables x-ray transmission measurements required for sample scanning and positioning. Finally, two-dimensional x-ray diffraction images are collected using a large format area detector (PILATUS 1 M-F, 450 μm Si sensor) with a maximum imaging frequency of 125 Hz and a required readout time of approximately 2.7 ms between images.

The combination of relatively high flux, high spatial resolution, and high imaging frequency make the GP table well-suited to some of the most challenging technical and scientific problems traditionally encountered in the extreme-conditions community (flux at the sample at 30.491 keV with the Si(111) monochromator and a 90 μm clean-up pinhole was estimated to be 8.3 × 10^11^ photons/sec/100 mA with a focus similar to that listed in the table above). HPCAT has focused on a few specific areas including; highest quality data from weak-scattering materials, highest spatial resolution to explore the heterogenous sample volume, and remote, programmable pressure and temperature control for time-dependent measurements.

**Fig. 3:**
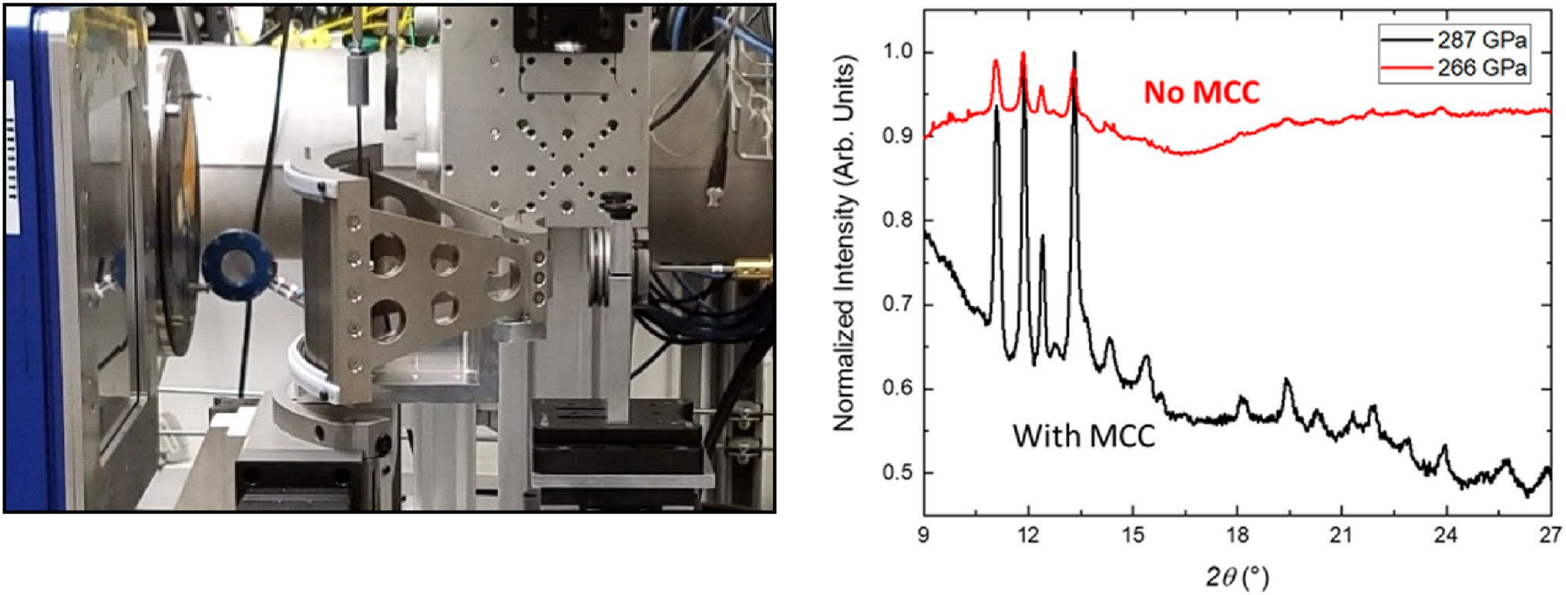
The multi-channel collimator setup is shown interspaced between the DAC and the Pilatus detector in the left panel. The diffraction patterns obtained from a sample above 260 GPa with and without the collimator are shown in the right panel

The minute sample volume characteristic of a DAC sample severely limits the available scattering volume, and this difficulty is exacerbated when studying low-Z materials with relatively few electrons, as is the case for many geosciences-relevant materials. The high flux at the GP table makes it possible to get high-quality data from weak-scattering materials (Fig. 3), with notable recent examples including the structure of hydrogen at multimegabar pressures (Ji et al. 2019) and the clarification of the phase diagram of lithium at pressures over a megabar and temperatures ranging from 220–400 K (Frost et al. 2019).

The combination of high flux with the high spatial resolution offered by the smaller beam focus enabled by the small KB mirror assembly makes the GP table an ideal instrument to study heterogeneous sample volumes. This has been instrumental for recent ultrahigh pressure studies for which there are large pressure gradients, often over a range of just a few microns. Rapid, high-resolution scanning of the sample volume reveals the highest-pressure region for studies involving toroidal anvils (Jenei et al. 2018) and nanocrystalline micro-anvils (Moore et al. 2018).

Remote, programmable pressure control—combined with high-frequency imaging capabilities—enables time-dependent studies using static high-pressure apparatus. A number of recent works, most notably exploring the stable and metastable structures of water/ice at modest pressures and low temperatures around water’s no-man’s-land (Lin et al. 2018; Shen et al. 2019), highlight the capability for such measurements at the general-purpose table.

A similar beam-delivering optics exists for bringing the monochromatic beam to the laser-heating table. Laser-heated diamond anvil cell (LH-DAC) coupled with the in-situ synchrotron x-ray diffraction is a unique and powerful experimental technique for studying a broad range of material properties under extreme conditions up to megabars of pressure and several thousand degrees Kelvin of temperature. Over the last decade, this technique has evolved into a routinely used and productive experimental method at synchrotron beamlines, leading to numerous major scientific advances and a large expansion of high-pressure research in physics, chemistry, geoscience, and materials science. One of the main applications of continuous wave laser heating (CWLH) has been the use of high temperature for overcoming kinetic barriers to phase transformation, and for enabling new materials synthesis at high pressure. Thus, technical developments have emphasized long-term system stability with heating duration in a typical experiment lasting from minutes to hours. Such long-term temperature stability of the CWLH has made possible many studies of phase transitions, materials synthesis, and sample annealing for equations of state (EOS) measurement. In recent years, modulated pulse laser heating is being increasingly used for high-pressure research. From a technical perspective, LH-DAC in a short time scale has several potential advantages. (1) It reduces the exposure of cell assembly to high-temperature conditions. This helps to maintain the cell assembly’s structure integrity and stability, thus improving the consistency and quality of experimental measurements and increasing the potential for reaching higher pressure and temperature. (2) The short heating duration helps to suppress thermally activated chemical diffusion and reaction (especially with the anvils). (3) Heating at short time scales and improved temporal resolution of temperature measurement have been very useful for high-pressure melting studies, and studies of phase transition dynamics under high pressure.

**Fig. 4:**
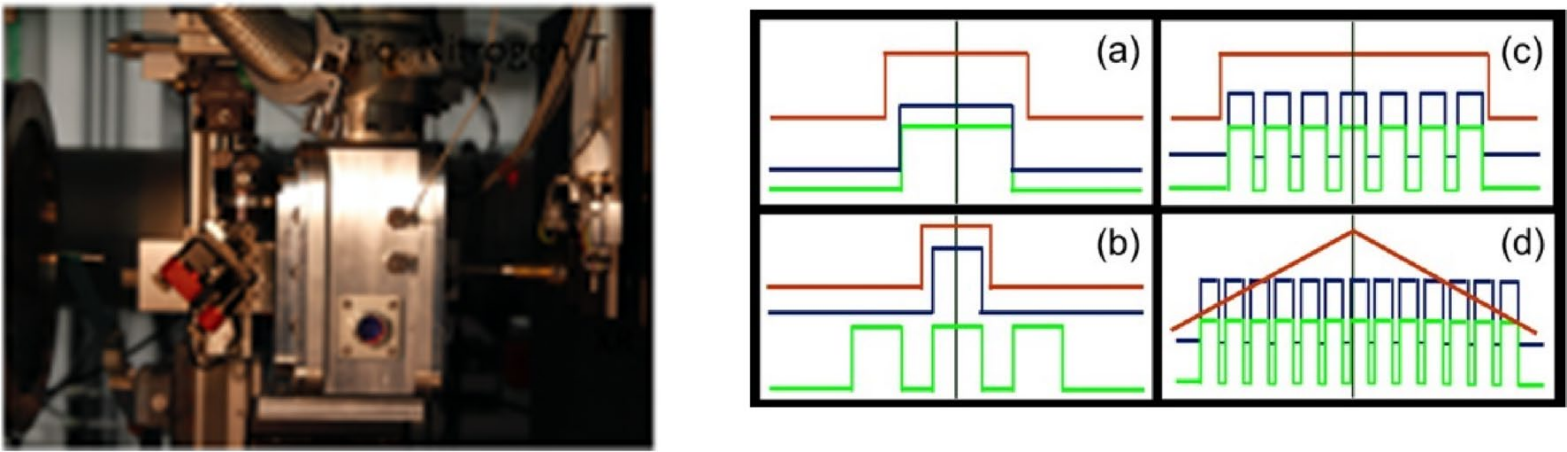
The photograph on the left shows the single-sided, pulsed laser heating setup used for synthesisizing hydride superconductors (Geballe et al. 2018) while the sample at megabar pressures is held inside an LN_2_ cryostat compatible for laser heating (Sinogeikin et al. 2018). Four-probe resistivity was measured using this setup while recording diffraction in-situ (Somayazulu et al. 2019). The set of oscilloscope traces on the right shows a few commonly used synchronizations of modulated pulse heating (cyan) with in-situ temperature measurement (green), and XRD measurements (magenta). Such a modulation allows us to measure and accumulate several diffraction patterns at elevated temperatures while at elevated pressures while ensuring the diamond anvils and/or the samples are not damaged. Details of such measurements on low-Z materials have recently been communicated (Kim et al. 2022)

The integrated system of LH-DAC with in-situ XRD, shown in Fig. 4 has been discussed in detail elsewhere (Meng et al. 2015). The laser heating system features double-sided heating in continuous-wave mode or in modulation mode and in-situ temperature measurements, independently on both sides. In modulation laser heating mode, laser power can be modulated into continuous ramp up and down, a single square pulse (microsecond time scale) or multiple pulses. Laser heating pulses can be synchronized with temperature measurement and with XRD measurements (Fig. 4) to perform pump-probe measurements. The laser heating temperature is measured using an imaging spectrograph with two area detectors, a back-illuminated CCD detector (PIXIS 400BR, Princeton Instruments) and a time-gated intensified electron-multiplying CCD detector (em-ICCD, PI-MAX4, Princeton Instruments), which can be used interchangeably. The spectral ranges available on the detectors are 169 nm (300 G/mm grating) and 340 nm (150 G/mm grating) on the CCD detector, and 157 nm (150 G/mm grating) on the em-ICCD detector. The em-ICCD detector can be electronically gated at the picosecond level and used for fast temperature measurement. In addition, the HPCAT laser heating setup has three unique features. First, the laser heating spot size can be varied in-situ and remotely from 4 to > 60 μm in the flat top area and from 9 to > 120 μm in FWHM (flat top area is defined as > 90% Gaussian laser peak intensity). Second, we use a mirror-pinhole setup at the entrance of the spectrometer, which allows the experimenters to observe from where temperature is measured relative to the total area of the heated spot for precise alignment and to ensure experiment data quality. The third is the integration of laser heating and cryostat cooling. These three enhancements have enabled a plethora of novel scientific studies that include melting studies (Hrubiak et al. 2018, 2017) and in the synthesis of novel super-hydrides synthesis and in-situ validation of record T_c_ in Lanthanum hydride superconductors (Somayazulu et al. 2019; Geballe et al. 2018).

16ID-B is the most sought-after beamline at HPCAT and while upgrade plans include enhancements, the priority is to bring this end-station online at the earliest. Upgrade plans include stabilizing the intensity and position of the focal spot in the end-station by replacing the first crystal mount of the branching monochromator. A pair of beam position monitors included in the FOE (16ID-A) and the end-station (16ID-B) will be used to maintain the beam position on the focussing optics while an accelerometer included with the first crystal is used for feedback. In addition, high-quality KB mirrors (with slope errors < 2.5 nm, RMS < 0.5 nm) are being procured for the large benders while pre-engineered KB mirrors are being evaluated for the small focal length option. The smaller slope errors and beam divergence due to the APS-U is expected to reduce the 90% width of the focused beam from the current 20 μm to 10 μm and even smaller with pre-figured mirrors, making ultra-high pressure measurements better. Optics simulations have suggested that these options will ensure tight focus and better beam profiles that will certainly enable better spatial discrimination and these simulations match our measurements very closely. A 2 M PILATUS detector with Cd-Se sensor, rencently received and being installed at the time of this report, will replace the existing detector to improve efficiency at higher operating energies. Plans include obtaining a high-speed, small format detector (> kHz frame rates) to perform a suite of time-resolved measurements on both tables in 16ID-B.

### Beamline 16-BM-D: general purpose table for robust x-ray diffraction experiments

16-BM-D experimental station is dedicated to micro-focused beam X-ray diffraction, X-ray absorption spectroscopy, and micro-tomography imaging for high-pressure research. The beamline utilizes a fixed-exit geometry of the monochromatic beam with a covered energy range of 6–60 keV (ΔE/E = 1.45 × 10^–4^) and a robust energy scanning capability with high fidelity energy calibration (Park et al. 2015). A variety of static compression experiments with diamond anvil cell can be conducted with resistive heating, cryo-cooling, radial geometry X-ray absorption, and parallel beam radiographic imaging measurements combined variously.

**Fig. 5:**
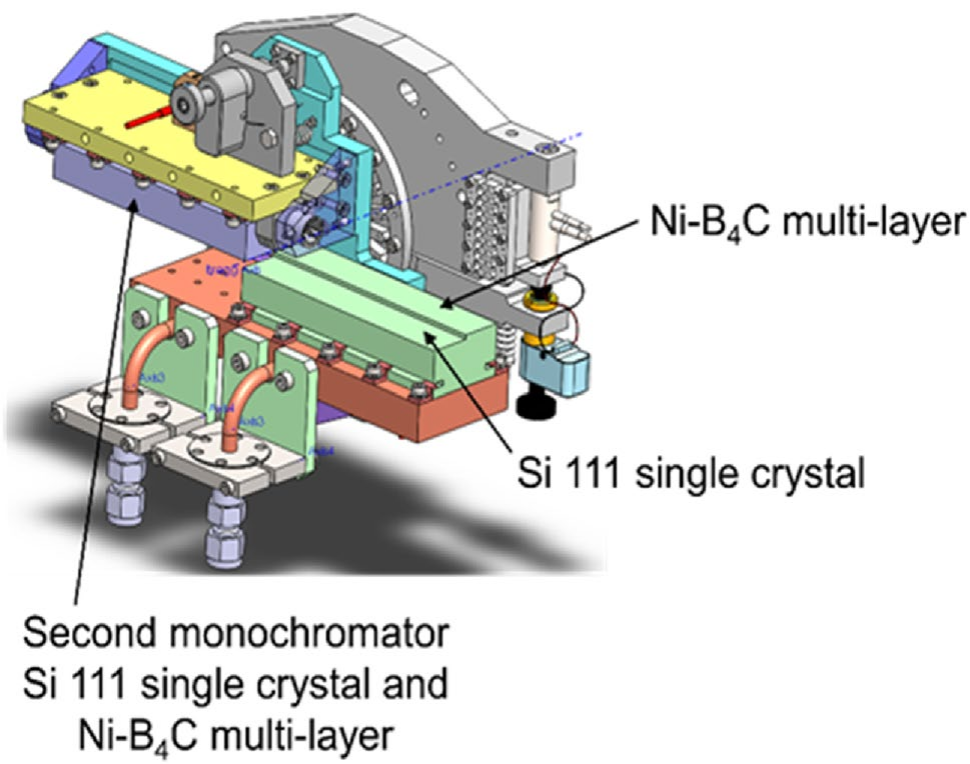
Schematic of the combined double crystal monochromator (DCM) and the double multilayer monochromator (DMM) mounted on a weak-link rotation stage. Both the monochromators share the same Si 111 single crystal substrates; the DMM portion of the first monochromator is stepped to permanently adjust the gap between the two monochromators; the DMM portion of the surface was super-polished before depositing the multilayers using modular deposition system at the APS^3^. The multilayer is composed of 300 layers of Ni-B_4_C bilayer yielding a d = 30 Å and γ = 1/3

The beamline has been very productive, however, there has been a constant demand for a higher incident flux to improve the measurement efficiency, especially, for tomographic imaging, time-resolved diffraction, and amorphous/liquid scattering. To enhance the incident flux without compromising the experimental capabilities, we recently introduced a high-throughput double multilayer monochromator (DMM) that allows dual-mode operation in tandem with the double crystal monochromator (DCM). The two monochromators share the same Si 111 single crystal substrate; therefore, a unique geometric shape has been designed to accommodate the largely different incident angles and the beam footprint for the two monochromators. A schematic of the combined DCM-DMM dual-mode monochromator is shown in Fig. 5. The specification of the DCM and its mount (Narayanan et al. 2008) remains the same as previous (Park et al. 2015); therefore, only the details of the DMM and its characteristics are described here.

The bilayer of the DMM is made of Ni-B4C with the unit spacing d = 30 Å and the asymmetric ratio 1/3. About 300 layers are deposited on a super-polished flat Si 111 single crystal substrate (metrology measurements on the silicon substrates (120 mm long and 21.75 mm thick) have indicated them to have a d space uniformity of 0.15%, RMS surface roughness of ~ 0.5 Å and an RMS slope error of ~ 0.6 μrad), using a modular atomic-layer deposition system at the APS (Conley et al. 2014). The corresponding bandwidth is 1.3%. The multilayer is deposited on the same Si 111 single crystal substrate that is used for DCM. The DCM portion is separately polished and etched to obtain a strain-free surface. The substrate of the first multilayer monochromator is stepped up by 1.6 mm from the DCM surface that has 2.2 mm gap between two crystals (see Fig. 5) so that the resultant gap between two multilayer monochromators is 0.6 mm. The second crystal shares the flat surface for both DCM and DMM. The center of rotation is located at the edge of the flat second monochromator. The tight gap between two multilayer monochromators is to achieve the desired energy range of 6–45 keV that matches the existing demand at the beamline but creates a large change in the beam footprints because of the shallow incident angles (corresponding to up to 110 mm of the crystal lengths). The beam offset for DMM is, therefore, maintained at about 1.2 mm, which is substantially different from 4.4 mm gap for DCM. Therefore, the downstream optics requires a systematic shift to compensate this difference when the setup is switched between the two monochromators.

**Fig. 6:**
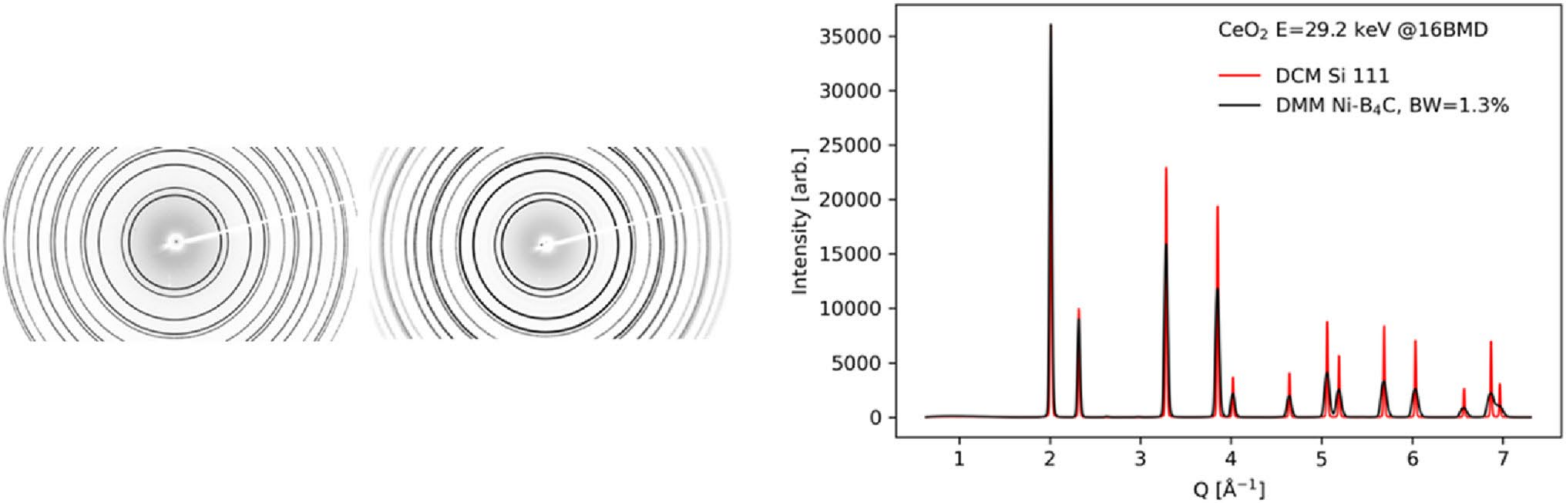
A comparison between CeO_2_ powder diffraction patterns measured at E = 29.2 keV with Si 111 double crystal monochromator (DCM) and Ni-B_4_C 300-layer double multilayer monochromator (DMM) at an identical diffractometer geometry. The two patterns are normalized with respect to the intensity of the first diffraction peak

The 1.3% bandwidth of DMM and the intrinsic multilayer reflectivity (R2 = 0.7–0.9 as the function of energy) delivers 70–90 × the DCM beam flux (Table. 2). The gain in the flux improves the measurement efficiency by nearly two orders of magnitude compared to that of DCM but at the cost of energy resolution. As an example, a direct comparison between powder diffraction patterns of CeO_2_ powders (NIST standard) measured with DCM and DMM realistically shows the gain and the cost (Fig. 6). The DMM beam has been successfully utilized for multiple user experiments including strength measurement using radial diffraction (Burrage et al. 2021), DAC-based high-pressure tomographic imaging (unpublished data), and a fast-2D scanning diffraction microscopy (unpublished data).

**Table 2:** Gain by DMM bandwidth relative to DCM

E (keV)	ΔE/E (DCM)	ΔE/E (DMM)	DMM R^2^	Relative gain
15	1.45 × 10^–4^	1.27 × 10^–2^	0.94	82
30	1.44 × 10^–4^	1.44 × 10^–2^	0.90	90
45	1.47 × 10^–4^	1.34 × 10^–2^	0.74	67

Encouraged by the large user base that benefits from the combined XAS-XRD measurements at 16BM-D, the upgrade plans for this end-station include retaining the current capabilities and redesigning the experimental table to also enable Laue diffraction and mulit-crystal diffraction (currently performed in BM-B station). The main modifications include an experimental table that will enable both these techniques with minimal switch-over time and both flipping the existing DCM/DMM to a horizontal configuration and also allowing the white beam pass-through. Details of the burgeoning Laue diffraction program are presented below.

### Beamline 16-BM-B: high-pressure laue diffraction for in situ structural deformation and transformation studies

Polychromatic X-ray diffraction is a powerful tool for investigating pressure/stress-induced microstructural changes accompanying plastic deformation, phase transitions, twinning, crystal growth, recrystallization and other physical or chemical process introducing substantial changes in single crystals. Using Laue diffraction, a similar number of reflections can be recorded one to two orders of magnitude faster compared to the most widely implemented monochromatic beam diffraction technique, while covering a larger swath of the reciprocal space. Therefore, Laue diffraction, implemented in-situ and operando, provides an overall improvement in time resolution and, as there is no need to rotate the sample, also much better spatial resolution compared to monochromatic x-ray diffraction. Currently, the polychromatic x-ray diffraction technique is implemented to characterize the mechanical behavior of materials under external stress (Cornelius and Thomas 2018) and to investigate mechanisms of pressure-induced phase transitions. At HPCAT, Laue diffraction is mainly used to study samples under compression in diamond anvil cells (DACs). An experimental setup specifically optimized for this purpose has been developed at the 16-BM-B beamline (Popov et al. 2015, 2019a, b). Studies under essentially hydrostatic compression are performed using noble gases as pressure transmitting mediums. For non-hydrostatic compression studies other pressure media, e.g. KBr, NaCl, are used. An important application of Laue diffraction to samples under non-hydrostatic compression is understanding the influence of the deviatoric stresses on the mechanical properties of materials at high pressures.

**Fig. 7:**
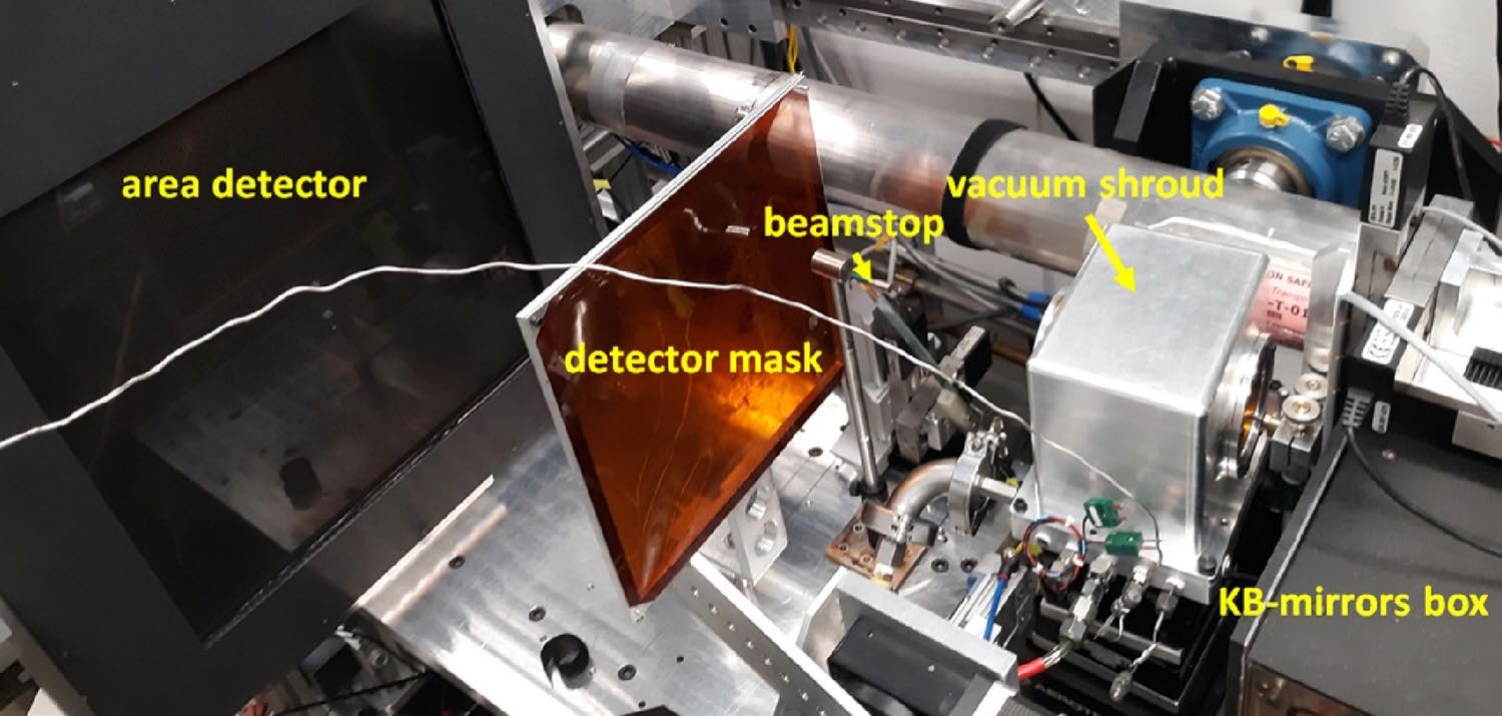
HPCAT Laue diffraction experimental setup to study samples under high pressure

Since the geometric opening of a DAC is limited, Laue diffraction is optimally performed in the transmission geometry (Fig. 7). The large X-ray energy range, with a high energy limit of about 90 keV, provides enough reflections for a fairly complete data set from a single-crystal. The X-ray beam is focused by KB-mirrors down to 2 μm^2^ at the sample position offering a high degree of spatial resolution. For measurements at high temperatures, an external resistive heater is used inside a vacuum shroud (Sinogeikin et al. 2015) to avoid oxidation of the DAC (Fig. 7) at temperatures in excess of 900 K. Ongoing efforts in the high-pressure community to introduce internal resistive heaters are also being adapted with our setup, with the goal of extending the range and control of temperatures. Application of internal resistive heaters can also substantially extend the limit of 2θ angles as the DAC assembly becomes more compact. The multi-position detector arm allows Laue diffraction measurements up to 90° detector geometry. This is useful for mapping residual strain in thin section samples or using panoramic DACs. For a given sample to detector distance, using a 90^0^ detector geometry increases the precision of deviatoric strain refined by about 10 times.

Preparation of DAC-sized samples from bulk materials by mechanical processing may introduce substantial changes to the initial micro-structure. To overcome this problem, a laser cutting machine (Hrubiak et al. 2015) in HPCAT sample preparation laboratory is used to cut samples having sizes appropriate for DACs. Positioning of the sample is also vital for Laue measurements, and with micro-manipulator available at HPCAT it makes it possible to position samples in the center of gasket holes and reduce diffraction signal from gasket material. A recent addition of the fs laser cutting system equipped with the Spectra-Physics SOLSTICE laser (5 mJ pulse energy, ~ 120 fs pulse width) for cutting samples is expected to improve sample preparation capabilities especially for fashioning micron-sized samples with no thermal shock damage. Recent measurements on zirconium test samples have shown that these samples have better overall integrity and are best suited for in-depth phase transition mechanism studies. There are various and ongoing experiments using the Laue diffraction setup at BM-B that are clearly demonstrating the power of this technique. Future move of Laue setup to BM-D during the upcoming upgrade, and combining with switchable mono-XRD, will further help optimize the power of Laue type measurements and attract a new user community to this capability.

With Laue, a typical data collection procedure is a series of two-dimensional (2D) translation scans conducted in-situ along with compression, which provides both time and spatially resolved microstructural information simultaneously. In general, the data analysis procedure consists of two stages (Popov et al. 2019b). Indices of reflections are determined first to identify single crystals present in the sample and subsequently determine their orientation. This is followed by building maps of reflections that provide information on crystal morphology. Finally, the deformation of single crystals is determined by the shape of diffraction spots and the variation of their respective positions across a crystal. The changes in orientation relations, morphology, and deformation of crystals, across a pressure-induced process, are observed in-situ and analyzed using the software, PolyLaue, developed inhouse by D. Popov (Popov et al. 2019b). It is also important to note that various analysis tools are also available and being adapted by the community to improve data processing – broader discussion of existing and in-development software tools requires much more detailed discussion and will be presented in elsewhere.

### Beamline 16-BM-B: large volume paris-edinburgh press program

At the experimental station 16-BM-B, capabilities for comprehensive high-pressure x-ray studies using a large volume press have been established. Many studies can greatly benefit from large sample volume, enabling multiple experiment types that are not easily feasible with small volume devices like the DAC. One particular area of interest is to investigate the relationship between the microscopic structure and the macroscopic properties of matter at high P–T conditions (Kono et al. 2014). The beamline 16-BM-B is equipped with a Paris-Edinburgh (PE) press (Klotz et al. 2004), integrated with a multitude of x-ray techniques and other optical and ultrasonic techniques for *in-situ* characterization at high P–T conditions. In addition to the *in-situ* characterization of materials properties, the large volume of PE press allows *in-situ* studies of materials synthesis (Iwan et al. 2021).

**Fig. 8:**
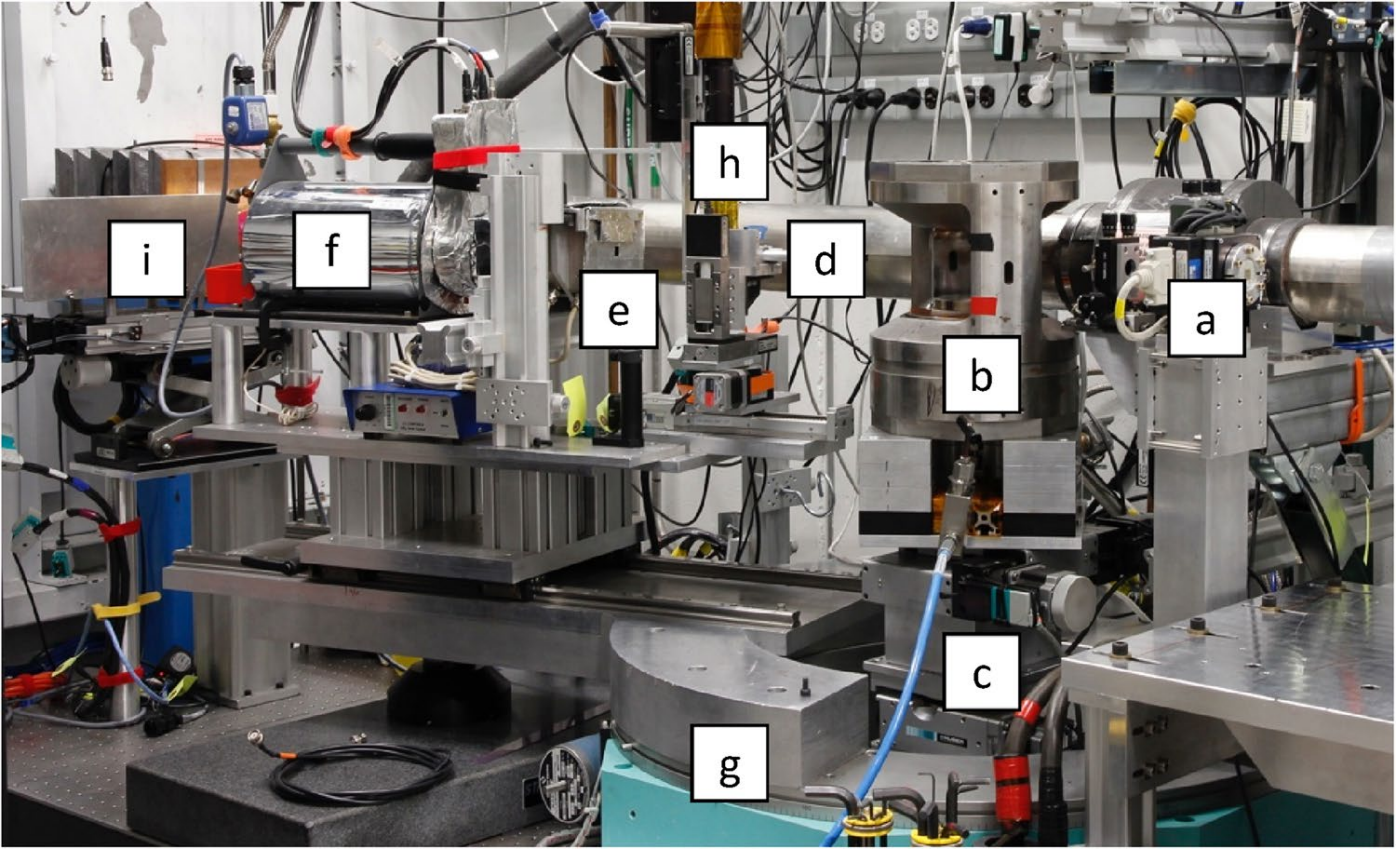
PE press setup at 16-BM-B: **a** collimating slits; **b** PE press; **c** positional scanning stages; **d** EDXD collimating tip; **e** EDXD detector-side collimating slits; **f** EDXD detector; **g** EDXD detector 2θ motion goniometer; **h** high resolution radiography system; **i** high speed radiography system

**Fig. 9:**
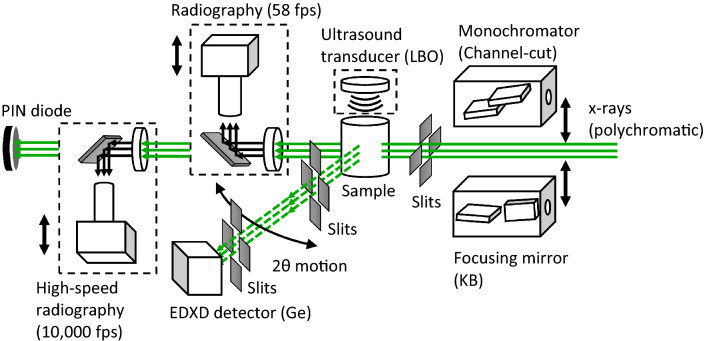
Beamline setup at the 16-BM-B PE press table. PE press and pressure generating mechanism are not shown

Benefitting from the broad energy range of the beamline and the large opening access of the PE press, the energy dispersive x-ray diffraction (EDXD) setup at the beamline covers a large momentum transfer (large-Q), allowing for the collection of structure factor data (q) between 1 and 30 Å^−1^. A large q coverage is essential for reliable structural information in particular of amorphous materials and melts (Kono et al. 2016, 2020b; Ohira et al. 2019; Shu et al. 2020b; Eastmond et al. 2021). PE press inside the 16-BM-B station and the schematic of the setup is shown in Fig. 8 and 9. Incident polychromatic x-rays can be either collimated with slits (parallel beam) or focused using KB focusing mirrors. A channel-cut monochromator is available for some specialized experiments (e.g. density measurement by absorption scanning). EDXD is collected using an HP-Ge energy dispersive detector at user-adjustable 2θ angles. Two scintillator-based radiography systems with a spatial resolution of better than 2 μm are available for normal operation with a frame rate of 58 frames per second (fps) and for high frame rate radiography up to 10,000 fps, respectively (Kono et al. 2014).

While the above x-ray characterization (EDXD, radiography, absorption) may be viewed as the base setup, other techniques for macroscopic properties, such as sound speed (Deng et al. 2019) (Jordan et al. 2021), elastic and plastic deformation studies, fluid viscosity, density, liquid (im)miscibility (Kono et al. 2015), and thermo-electric properties (Baker et al. 2021) can be implemented as requested. The established characterization techniques (Kono et al. 2014) include ultrasound echo, falling sphere viscometry, monochromatic x-ray absorption scanning, phase contrast radiography, and specialty sample cells with electrical probes (Baker et al. 2021). Using the PE press, samples can be simultaneously compressed and heated. The typical P–T range for the samples in the PE press, with dimensions up to several millimeters, is up to 7 gigapascal (GPa) and 2300 K. Table 3 lists the P–T ranges corresponding to various cell designs that are available for user experiments.

**Table 3:** Cell assemblies available with PE press and relative P–T ranges that can be achieved

Type of cell	Sample dimension (mm^3^)	Maximum pressure (GPa)	Temperature coverage (K)	Note
Standard type	6	7	300–2300	Ultrasound up to 1600 K
Cupped Drickamer Toroidal (CDT)	1	13	300–2300	
Double stage	0.08 (0.1 mm × 0.8 mm^2^)	100	300–800	Based on a culet size of 0.8 mm diameter

### New developments

#### Remote-controlled large-volume pressurization system

Due to the COVID-19 pandemic, we have introduced a remote operations mode option starting July 2020 and ongoing. In the remote operations mode, the full control of experimental parameters and data collection is turned over to the remote beamline users. Furthermore, what has enabled the use of PE press for remotely controlled experiments is the newly installed dual-syringe pump at the beamline 16-BM-B. Precise hydraulic control of the oil pressure—used to drive the PE press was previously achieved by a manual pumping system which excluded the possibility to perform remotely controlled experiments. The new motorized syringe pump system can reach pressure set-points faster than the previously used manual pumps—greatly improving the beamtime use efficiency. In addition, the system offers exceptional low-flow stability, which translates to an improved stability for the PE press and maintaining the sample pressure.

#### Ultrasonic

Sound velocity can be highly sensitive to atomistic structural changes in material, thus integrating sound velocity measurement and x-ray diffraction-based structural measurements at HPCAT has provided information for an in-depth physical understanding of materials’ properties (Kono et al. 2014; Deng et al. 2019; Jordan et al. 2021). Conceptually, the ultrasound transducer is attached behind the top anvil of the PE press that generates and receives elastic waves (Fig. 1, pressure generating mechanism, anvils and PE press are not shown). The sound wave travel times are obtained by correlating echoes on the transducer return signal, which is acquired by a digital oscilloscope, while the traveled-distance of the wave through the sample is determined by *in-situ* x-ray radiography. The sound velocity measurement technique, coupled with the PE press, and *in-situ* x-ray diffraction and radiography at 16-BM-B (Kono et al. 2014), has recently undergone several additional improvements:

- Signal acquisition electronics (upgraded signal conditioning amplifier and signal gating).
- Software for automation of experiment workflow (new in-house GUI-driven software).
- Ultrasound data reduction and analysis (Sturtevant et al. 2020; Jordan et al. 2021).
- Specialized sample containment (Jacobsen and Velisavljevic 2015).

The improvements have resulted in an increased ultrasound echo signal level (5–10 × improvement), efficiency (data collection time from minutes to down to seconds), and throughput (ability to study more samples per beamtime) of visiting user experiments. Further developments for enhancing the resolution of the system are ongoing and shall be reported elsewhere.

#### Double stage compression

Large sample volume in the PE press allows for accommodating additional two opposed diamond anvils as second-stage anvils. We have successfully used the double-stage configuration for amorphous structural measurements at ultrahigh pressures in the Mbar range (Kono et al. 2016, 2020a, b) (Shu et al. 2020b). Culet sizes of the second-stage diamond anvils are typically chosen from 1.2 mm to 0.6 mm in diameter, covering pressure ranges up to 60 GPa and 150 GPa, respectively. Currently, the temperature is still limited up to 800 K, above which the second stage diamond anvils start to yield causing reduced pressures. Similar to the DAC, a pre-indented gasket is used with a central hole as a sample chamber. The double stage configuration provides a much larger sample volume, 0.3–0.6 mm in diameter and 0.1–0.3 mm in thickness, than that in the conventional diamond anvil cell, but still with a large pressure coverage over 100 GPa, which is particularly useful for studying weak scattered samples at ultrahigh pressures. The large sample volume not only makes it possible to detect weak scattered signals but also provides needed space for effective collimation to suppress the unwanted background signals from surrounding materials. Recently, the double-stage PE press assembly has been used for measuring structure factors and pair distribution functions of glasses and melts in the Mbar pressure region (Kono et al. 2016, 2020b; Ohira et al. 2019; Shu et al. 2020b).

The PE press and the integrated x-ray and other optical and ultrasonic techniques have been widely used for addressing problems in multidisciplinary fields. In recent research activities, elasticities of materials have been determined using simultaneous structural probes and sound speed measurements (Kono et al. 2015; Sturtevant et al. 2020; Jordan et al. 2021). Viscosities of geologically relevant melts have been measured for understanding the dynamics of the Earth’s interior (Kono 2019) (Stagno et al. 2020) and densities of melts provide essential information on the structures of planetary interiors (Deng et al. 2019; Mouser et al. 2021). Structures of silicate and oxide glasses have been measured to high pressures at pressures above 100 GPa (Ohira et al. 2019; Shu et al. 2020b; Kono et al. 2020b; Hu et al. 2021). High cation coordination is systematically observed in dioxide glasses with increasing pressure, showing plateaus at 4-, 6-, and 9-coordinated networks (Shu et al. 2020b).

Another example of PE capabilities is demonstrated by recent measurements of the structure of liquid lithium at high pressure (Shu et al. 2020a). Major challenges in experimentally determining the structure of liquid lithium include the weak signals in X-ray scattering and its inherent chemical reactivity. Prior to the recent work on the PE press at 16-BM-B at HPCAT, the structure of liquid lithium was only measured at ambient pressure. The large sample volume in the PE enabled the detection of the scattering from liquid lithium. By using a LiF cylindrical capsule, liquid lithium was found to be chemically intact. The structure of liquid lithium was successfully measured under an isothermal compression path covering a pressure range from 2 to 11.5 GPa at a temperature of 600 ± 30 K. A structural change from bcc-like to fcc-like local ordering was observed at pressures around 7.5–8.7 GPa. The observed structural changes in liquid lithium were shown to be consistent with the experimentally determined melting curve of lithium (Schaeffer et al. 2012).

The changes in the magnetic lattice that accompany the APS-U affect our BM beamlines more than the ID beamlines. The presence of two sources precludes us from using the two ends of the fan as we are currently operating. Plans needed to be made to run all the above three programs that have a large user base and optimizing them best for tandem operations which would eat up 50% of the beam time in each technique. Accordingly, we have planned to install a large 1 m mirror in 16BM-A to harvest a larger horizontal beam for the PE press. This is expected to increase the data collection efficiency by a factor of 10 and would especially benefit diffraction measurements (both crystalline and amorphous measurements). Similarly, the diffraction measurements planned in 16BM-D (both techniques) will be enhanced by the addition of faster detectors with more efficient sensors (CdTe and GaAs). The future of the PE cell program, the white beam Laue program and the XAS-XRD program at the BM lines of HPCAT are therefore being enhanced and tailored to meet the growing needs of NNSA mission-driven goals and the wider general user community.

## Beamline support equipment

### Remote pressure control

The conditions in the sample environment are crucial to successful experimental measurements. HPCAT maintains a variety of devices for controlling sample pressure and temperature in a DAC. Due to the time inefficiencies involved with opening the experimental hutch to directly access the sample, most of these devices allow for remote control of the pressure, temperature, or both. Control of pressure is the driving motivation for using a DAC and efficient use of time is a necessary component of synchrotron experimental management. Remote sample pressure control addresses both issues and allows users of HPCAT facilities to control the pressure quickly and reliably, on their samples.

For room temperature measurements, HPCAT equips each station with stepper motor-driven gearboxes that can accommodate the popular Princeton symmetric cell, as well as any other DAC with a 1.5″ diameter bolt circle for its pressure screws. In addition, each hutch has a PACE 5000 gas pressure controller that is used to inflate a double-diaphragm which, in conjunction with canisters designed at HPCAT, can increase pressure in a wide variety of DAC designs. For several popular cell types, an in-house designed pusher plate/pin system can also decrease cell pressure, allowing experimenters to cycle pressure with control in both directions (i.e. compression and decompression studies) (Sinogeikin et al. 2015). More rapid cycling of pressure in a symmetric cell can be achieved through a combination of a double-diaphragm (either for compression or decompression) with a piezoelectric driver (Evans et al. 2007; Smith et al. 2015). This arrangement allows the initial sample pressure to be set by the diaphragm pressure and the amplitude of the cycle to be determined by the piezo voltage. In a similar fashion, a step-wise pressure increase or decrease can be induced in the cell.

### Remote temperature control

In addition to the laser heating technique discussed in previous sections, additional capabilities are available at HPCAT for tunning the temperature environment in a DAC via whole-cell heating with resistive heaters or by cooling in one of several different cryostat designs.

For mild heating (up to ~ 200 °C) an array of copper and brass block heaters machined to fit popular DAC models are available. Higher temperatures require the cell to remain under a vacuum while heating to protect it from oxidization damage. Two different vacuum heating furnaces have been developed for our users, one which uses several cartridge heaters in a copper block which is clamped onto the cell, and a second design in which a coiled heater fits directly around the circumference of the DAC. Both designs feature integrated diaphragm pressure control (bi-directional for the coil heater) and can be used with several different DAC designs. Each of these vacuum heaters can heat to temperatures in excess of 600 °C and allow for a range of x-ray measurements to be performed at these conditions. Typical temperature excursions are held below ± 5^0^ C with the external block heaters but can be maintained within ± 1^0^ C when enclosed in a vaccum shroud.

All of our available cryostats feature a near vibration free, open loop cooling scheme in which the cryogen flows either over the surface of the DAC or through a channel inside a copper block that is clamped to the DAC. The double wall, vacuum insulated design required for the former design limits its usefulness for certain techniques, but allows for the lowest base temperature (~ 4.2 K when used with helium cryogen) and can accommodate essentially any type of DAC with either gearbox or diaphragm pressure control. Two cryostats with the cooled copper block design can be configured for many types of DACs by simply swapping out the copper clamp based on the diameter of the DAC to be used. These cooling heads integrate bi-directional diaphragm pressure control for symmetric cells, or controlled pressure increase for other types of DACs, and allow for cell temperatures down to 12—15 K, with stability of ± 2^0^ K, if required. A Lakeshore 336 controller maintains temperature setpoints via a cartridge heater in the copper block and is accessible through a GUI on the control computer at each of our experimental end-stations.

### Sample preparation

While all users are encouraged to prepare their DACs before arrival at HPCAT to optimize x-ray beam use, it is inevitable that some loadings will fail during an experiment. For these instances, in addition to x-ray end-stations, HPCAT also maintains a well-stocked sample preparation lab to allow for quick sample re-loads or other modifications. The offline laboratory has three stereo microscope loading stations for manual sample loads, as well as a Microsupport Axis Pro SS micromanipulator for loading of complicated sample configurations. In case of air-sensitive samples and appropriate handling, a glove box (argon atmosphere) equipped with an Evo Lynx eyepiece-less stereo microscope is available. Since many inelastic scattering, XANES and tomography experiments require beryllium to be used as the gasket material, the sample lab fume hood contains an additional microscope dedicated to loading cells with Be gaskets. Gaskets of all materials can be drilled by the HPCAT laser drilling machine (Hrubiak et al. 2015). Most steel and rhenium gaskets are cut in air, however, Be gaskets are cut inside a special canister to contain dust and other materials and under an argon atmosphere to reduce oxidation during the cutting process. Even though most drilling is carried out on the laser cutting machine, some specialty applications still work best with older techniques, and in those cases a Hylozoic Products Micro EDM system and a Minitool mechanical drilling machine is also available.

Many of the experimental support facilities introduced over the years have been developed by staff and, as with any scientific work, in collaboration with our users. HPCAT is continuously looking at additional techniques/capabilities to support the evolving array of experimental research.

### Other off-line capabilities

In addition to the above, there are several offline capabilities that are also widely used. Each end-station is equipped with an online ruby fluorescence measurement system but two off-line systems that allow pressure measurement also exist. One of these is the Raman system equipped with a 532 nm (max. power 2 W) excitation that is equipped with super-notch filters and a high degree of spatial filtering. This allows the user to estimate the pressure (and the pressure distribution) from a measurement of the diamond Raman signal. Both these systems are calibrated against the beamline sample motors to enable a quick translation of the sample coordinates. In addition, off-line optical spectroscopy facilities include a multi-wavelength Raman spectroscopy system equipped with 532 nm, 488 nm and 660 nm diode lasers and a switchable set of filters to perform high-resolution measurements. This system also allows one to incorporate a cryostat (as also the 532 nm system described above) to perform in-situ pressure and Raman measurements (while simultaneously performing transport measurements, for example). Indeed, this capability to perform off-line transport and Raman measurements has been leveraged to discover several new pressure and temperature-induced transformations (Watkins et al. 2022) (Zhu et al. 2020). These discoveries were also aided by the off-line CO_2_ laser heating capability (Smith et al. 2018).

Transport measurements play a key role in both on-line and off-line characterization of melting transitions, structural transitions, and superconducting transitions. At HPCAT, these can be performed both with dc and/or ac setups. These can be incorporated with cryostats, dynamic DACs, RHDACs and coupled to small magnetic fields with designer anvils and/or external coils. With the recent excitement of hydride superconductor discoveries and the need for magnetic signatures, plans include enhancing these capabilities to also perform transport measurements with modulated magnetic fields that can detect small signatures and possibly in-situ. Specialized non-magnetic cells for these measurements have also been designed and are being tested in collaboration with researchers from the National High Magnetic field Laboratory at Tallahassee.

HPCAT has also been active in designing new classes of diamond cells to meet the evergrowing needs of techniques coming online. Diamond anvil cells tailored for magnetic measurements, tomography measurements, radiography measurements, IXS measurements, dynamic measurements adapted for flow type cryostats, external and internal resistive heating continue to be developed and made available to users. Details of these developments are transmitted through our webpages, hands-on workshops, and publications.
